# Nanotopographical Coatings Induce an Early Phenotype-Specific Response of Primary Material-Resident M1 and M2 Macrophages

**DOI:** 10.3390/ma13051142

**Published:** 2020-03-04

**Authors:** Tobias Schmitz, Maren Jannasch, Tobias Weigel, Claus Moseke, Uwe Gbureck, Jürgen Groll, Heike Walles, Jan Hansmann

**Affiliations:** 1 Department Tissue Engineering and Regenerative Medicine (TERM), University Hospital, 97070 Würzburg, Germany; Maren.Jannasch@uni-wuerzburg.de (M.J.); tobias.weigel@uni-wuerzburg.de (T.W.); Jan.Hansmann@uni-wuerzburg.de (J.H.); 2 Translational Center Regenerative Therapies (TLC-RT), Fraunhofer Institute for Silicate Research ISC, 97070 Würzburg, Germany; 3 Institute for Biomedical Engineering (IBMT), University of Applied Sciences Mittelhessen (THM), 35390 Gießen, Germany; claus.moseke@lse.thm.de; 4 Department of Functional Materials in Medicine and Dentistry (FMZ), University Hospital, 97070 Würzburg, Germany; uwe.gbureck@fmz.uni-wuerzburg.de (U.G.); juergen.groll@fmz.uni-wuerzburg.de (J.G.); 5 Core Facility Tissue Engineering, Otto von Guericke University, 39106 Magdeburg, Germany; heike.walles@ovgu.de

**Keywords:** nanotopographical surfaces, combination of physical vapor deposition and electrochemical etching, defined humanized test system, inflammatory response

## Abstract

Implants elicit an immunological response after implantation that results in the worst case in a complete implant rejection. This biomaterial-induced inflammation is modulated by macrophages and can be influenced by nanotopographical surface structures such as titania nanotubes or fractal titanium nitride (TiN) surfaces. However, their specific impact on a distinct macrophage phenotype has not been identified. By using two different levels of nanostructures and smooth samples as controls, the influence of tubular TiO_2_ and fractal TiN nanostructures on primary human macrophages with M1 or M2-phenotype was investigated. Therefore, nanotopographical coatings were either, directly generated by physical vapor deposition (PVD) or by electrochemical anodization of titanium PVD coatings. The cellular response of macrophages was quantitatively assessed to demonstrate a difference in biocompatibility of nanotubes in respect to human M1 and M2-macrophages. Depending on the tube diameter of the nanotubular surfaces, low cell numbers and impaired cellular activity, was detected for M2-macrophages, whereas the impact of nanotubes on M1-polarized macrophages was negligible. Importantly, we could confirm this phenotypic response on the fractal TiN surfaces. The results indicate that the investigated topographies specifically impact the macrophage M2-subtype that modulates the formation of the fibrotic capsule and the long-term response to an implant.

## 1. Introduction

Any medical device, prosthesis or biomaterial creates a trauma following implantation, whereby the presence of the implant subsequently effects the healing of the trauma site. The altered healing process is known as the foreign body reaction (FBR) and results in the worst case in a complete implant rejection [1]. Thus, the FBR is a key factor in the long-term survival and function of an implanted biomaterial [2]. During the FBR, macrophages play a major role [3,4]. Over time, an initial population of short-lived pro-inflammatory M1 macrophages is replaced by long-vitae M2 macrophages. The chronic accumulation and fusion of these M2 macrophages in the proximity of the implant induces the production of a dense fibrous capsule by fibroblasts, isolating the foreign body from the native tissue [4]. 

The FBR is known to be affected by surface properties such as implant topography and chemistry. Here, the interaction between defense cells and especially structures in the nanoregime has gained increasing interest [5,6,7]. The generation of a surface comprising nanofeatures is, in this case, particularly interesting for an extensively used biomaterial like titanium, as can be derived from the variety of manufacturing methods that are applied for this purpose [8,9,10]. A comparably cost-efficient method to generate oriented nanostructures on a large scale is the fabrication of nanotubular surfaces by electrochemical anodization of Ti. Nanotube (NT) arrays were already tested in biomedical applications, demonstrating that these surfaces bear potential in drug delivery, biosensing or surface-modified implants [11,12,13]. An approach to increasing the application domain for nanotube structures is to treat Ti coatings instead of bulk material [14]. It was found that electrochemical anodization is applicable for samples that are coated by physical vapor deposition techniques, e.g., direct current (DC)-sputtering, radio frequency (RF)-sputtering, electron-beam evaporation, or arc evaporation [15,16,17,18,19]. By a combined surface treatment composed of coating and anodization, the surface of relevant implant materials, such as CoCrMo-alloys could be modified [20]. Thereby, these materials were equipped with a corrosion-resistant, biocompatible, and nanostructured layer that additionally prevents the release of toxic ions from the underlying substrates [21,22,23].

Until now, the immunological response to a nanotubular-structured implant has mostly been investigated with bulk Ti. Ainslie et al. studied the inflammatory response of human monocytes on nanotubes with a diameter of about 80 nm, and could find that establishing a nanostructure on the surface of a Ti sample significantly reduces inflammation [24]. Moreover, nanotubular topographies are known to trigger differentiation and polarization of human monocytes into M1 or M2 macrophages depending on nanotube diameter [25]. Small nanotube diameters promote M2 polarization, whereas large nanotube diameters induce polarization towards M1 phenotype. In order to investigate the influence of nanotube diameter on the inflammatory response, murine macrophages were cultured on nanotubes with different diameters ranging from 30 to 100 nm [26]. Thereby, it was observed that TiO_2_ nanotube surfaces have an increased ability for quenching nitric oxide (NO) compared to the conventional control surface. Generated by macrophages in the wake of their natural immune response, NO subsequently causes a number of inflammatory signaling events. The comparative study revealed that nanotubes with a diameter of 70 nm have the strongest effect on the used animal-derived macrophages. A decreased NO activity in the presence of a nanotubular surface was also observed in vivo [27], whereby the nanostructured implants (nanotube diameter 100 nm) inserted into the abdominal wall of rats resulted in a reduced fibrotic capsule thickness for structured implants, compared to flat Ti surfaces. It was concluded that the catalytic TiO_2_ activity is enhanced by the higher surface area due to the presence of the nanotubes. In summary, all currently available studies on the immune-regulatory effects of TiO_2_ nanotubes indicate that introducing a nanotubular topography onto an implant surface modulates the behavior of macrophages in the FBR. Although this promising effect has been identified, the specific impact of nanotubular surfaces on each macrophage phenotype has not been elucidated. Due to the distinct roles of each macrophage phenotype during the inflammatory response to an implant, such information would enable us to gain a mechanistic understanding to further improve the nanotubular surfaces to control the FBR. Moreover, all previous studies are either, based on monocytes, macrophages of non-human origin, or on implantation of samples into animals impeding a direct correlation to humans. To establish a reliable in vitro assay, we tested the influence of different nanotube diameters on primary human macrophages with either M1 or M2 phenotype. This experimental approach is also strengthened by the fact that monocytes in vivo differentiate immediately after passing the endothelial barrier and entering the surrounding tissue [28]. 

In order to strengthen our results, the experimental procedure was employed to a second set of materials, whose topography could be varied gradually from smooth to different degrees of nanotopography, meaning that the style of the nanostructures is maintained, but their size can be varied. As the variation of anodization voltage in the case of nanotube fabrication allows the control of the resulting nanostructure, in the case of titanium nitride (TiN) coatings prepared by PVD, the process parameters, such as substrate temperature, amount of nitrogen in the process gas, or the deposition time represent adjustable process parameters to control the evolving nano topography [29,30]. TiN has a long history in the field of biomaterials, and its various forms have found applications ranging from protective coatings for orthopedic implants to electrodes for nerve stimulation or pacemakers [31,32,33]. Although, there is literature that investigates the in vivo encapsulation of TiN showing a fractal nanotopography, but focused on the inflicted change of electrical parameters due to the fibrous encapsulation, there are no studies that investigate the response of macrophages to this kind of surface structure, and thus, it represents an interesting opportunity for extending the scope of this study and verifying a subtype-specific response of macrophages to nanostructured surfaces [34,35]. 

The aim of this study was to examine the interaction of two different polarizations of macrophages with a carefully selected, manufactured and characterized variety of smooth and nanostructured surfaces, thus searching for significant differences in the respective cell surface interactions. The study of these interactions might give further insight about the future design of implant surfaces whose purpose is the support or impediment of a specific cell polarization or an associated reaction.

## 2. Materials and Methods 

### 2.1. Substrate Preparation

The glass slides that were used as substrates (Icefrost 76 × 26 × 1 mm³, Menzel, Germany) for the sputtering process were cleaned in subsequent 10-minute ultrasonic baths (Bandelin electronic, Berlin, Germany) with acetone, isopropanol and ultrapure water and were finally dried using nitrogen gas.

### 2.2. Physical Vapor Deposition (PVD)

As previously described in [36], the deposition of pure Ti films was done in a vacuum chamber equipped with a magnetron sputtering system and substrate heating. At least 1 h before the deposition, the glass slides were heated to a temperature of 200 °C. Argon was used as sputter gas and a multi gas controller (MKS Instruments, Andover, MA, USA) controlled the flow rate. During deposition, the flow rate was set to 137 sccm for all experiments, which resulted in a pressure of 4.0 × 10^−3^ mbar. A 13.56 MHz RF generator (RF 1000, Hüttinger, Freiburg, Germany) was used for magnetron sputtering and was operated at a power of 400 W. The sputtering time was 4 h, thereby creating films with thicknesses of approximately 4 µm. Based on deposition time and film thickness, for sputtering powers of 400 W the deposition rate was calculated as 0.25 nm/s. 

For the generation of nanorough TiN coatings, glass slides were first coated for 30 minutes with a layer of Ti with a sputtering power of 500 W and Ar flow rate of 137 sccm, without external heating. The nanorough TiN was then prepared in a gas mixture of Ar 137 sccm and N_2_ 2.0 sccm with the RF generator operated at a sputtering power of first 800 W for 2 minutes and 500 W for 15 (TiN 15) or 60 (TiN 60) minutes afterwards.

Smooth TiN coatings were prepared on a heated substrate holder with a temperature of 200 °C. After depositing a layer of Ti using the same process parameters as for the nanorough coatings, a flow of 2 sccm N_2_ was added to the process gas, and the samples were coated with a sputtering power of 800 W (2 minutes) and subsequently 600 W (18 minutes). A summary of the process parameters is presented in Table 1.

### 2.3. Electrochemical Anodization

As previously described in [36], the PVD films were etched for 1 min in diluted hydrofluoric acid (0.5 wt %) directly before placing the samples in the electrolyte. The distance between the Pt-coated stainless steel plate which acted as counter electrode and the working electrode was 2 cm, whereby the homogeneity of the electrolyte was ensured by magnetic stirring. The Ti films were anodized for 2 h using a DC power supply (Voltcraft VLP 1602 Pro, Conrad Electronic AG, Wollerau, Switzerland) and a mixture of phosphoric and hydrofluoric acid (1 M H_3_PO_4_ + 0.2 wt % HF) [37]. The as-anodized samples were directly rinsed with ultra-pure water, prior to a subsequent 15-minute ultrasonic bath in ultrapure water to remove all residual electrolyte and a final drying step using compressed air.

Annealing of the as-anodized nanotubes was performed in a high power furnace (L08/14 Nabertherm, Lilienthal, Germany) in air. The target temperature of 450 °C that was reached after a heating time of one hour was maintained for 3 h with a subsequent cool down phase of at least 4 hours.

### 2.4. Coating Characterization

#### 2.4.1. Morphology and Surface Roughness

The surface morphology of all samples was determined by scanning electron microscopy (Zeiss CB 340, Oberkochen, Germany) and atomic force microscopy (Nanosurf FlexAFM, Nanosurf GmbH, Langen, Germany). The average values of the inner diameters obtained by analysis of scanning electron microscopy (SEM) images of the different nanotube surfaces are arithmetic mean values of 20 nanotubes per specimen. For the analysis and quantification of surface roughness, the standard atomic force microscopy (AFM) instrument software Nanosurf Easyscan 2 was applied. The line roughness values of three different positions on each sample were analyzed with scan field sizes of approximately 5 µm × 5 µm. The measurements were performed in air under tapping mode, using a scan speed of 0.3 s/line, and a silicon cantilever Tap190-Al G (Innovative Solutions Bulgaria Ltd, Sofia, Bulgaria) with a tip radius of about 10 nm or smaller [36].

#### 2.4.2. X-ray Diffraction

The crystallographic properties of the samples were determined by X ray diffraction (XRD) using a D5005 X-ray diffractometer (Bruker AXS, Karlsruhe, Germany) with grazing angle geometry (angle of incidence 2°) [30]. The diffractometer used Cu-K radiation with a voltage of 40 kV and a tube current of 40 mA. The diffraction patterns recorded in a 2Θ range from 20–80° were evaluated with the software DiffracPlus EVA (Bruker AXS, Karlsruhe, Germany) and compared with reference patterns from the JCPDS database [38].

#### 2.4.3. Contact Angle

The analysis of surface wettability was carried out with a contact angle measuring device (OCA 15EC, Dataphysics, Filderstadt, Germany). Directly after preparing the samples, they were cleaned with isopropanol and dried by compressed air. Then, three droplets of 3 µL ultrapure water were dripped onto different parts of the surface of each sample.

### 2.5. Ethical Clearance Statement

Primary cells for studies addressing cell-biomaterial interactions were isolated from human peripheral blood under informed consent according to ethical approval granted by the institutional ethics committee of the Julius-Maximilians-University Wuerzburg (vote 182/10). Furthermore, procedures were in accordance with the Helsinki Declaration of 1975, as revised in 2008.

### 2.6. Isolation and Differentiation of Human Monocytes to Macrophages

Mononuclear cells from peripheral blood (University Hospital Wuerzburg, Germany) were isolated according to a previously published protocol [4]. Briefly, following ficoll gradient centrifugation (GE Healthcare, Freiburg, Germany), monocytes were separated by a negative magnetic cell separation (MiltenyiBiotec, Bergisch Gladbach, Germany) to remove T cells, NK cells, B cells, dendritic cells, and basophils. Obtained monocytes (purity 90 ± 4%) were cultured in RPMI GlutaMax (Gibco, Carlsbad, CA, USA) plus 10% fetal calf serum at a concentration of 1 × 10^6^ cells per ml and a density of 1.5 × 10^5^ cells per cm^2^ on standard polystyrene cell culture dishes (TPP, Trasadingen, Switzerland). 

By supplementation of recombinant human granulocyte macrophage colony-stimulating factor (GM-CSF, Peprotech, Rocky Hill, NJ, USA) at a concentration of 40 ng per ml for 6 days, differentiation to the M1 phenotype was triggered. Differentiation of monocytes to the M2 phenotype was induced by supplementation of 40 ng per mL recombinant human macrophage colony-stimulating factor (M-CSF, Peprotech) for the same timeframe. Respective medium in each differentiation experiment was exchanged on Day 3. The cells were harvested by mechanical cell scraping on Day 6. 

To ensure the required phenotype, expression of differentiation cluster proteins was characterized in flow cytometry. The expression profile was evaluated by antibody staining of 2 × 105 macrophages per antigen. CD14 (555-397, BD Bioscience, Heidelberg, Germany), CD68 (11-0689-42, eBioscience, Frankfurt am Main, Germany), and CD206 (12-2069-42, eBioscience) were used as positive markers for macrophages. Markers CD80 (12-1639-42, eBioscience) and CD163 (17-1639-42, eBioscience), typically inversely-expressed by M1 and M2 macrophages, were used to proof differentiation towards the required phenotype. The samples were analyzed in a FACS Calibur flow cytometer (BD Biosciences) and data were further processed in FlowJo software (Tree Star, Ashland, OR, USA). The biological variability was covered by using cells isolated from five donors in the respective experiments.

### 2.7. In Vitro Testing of Biomaterials

**Figure 1:**
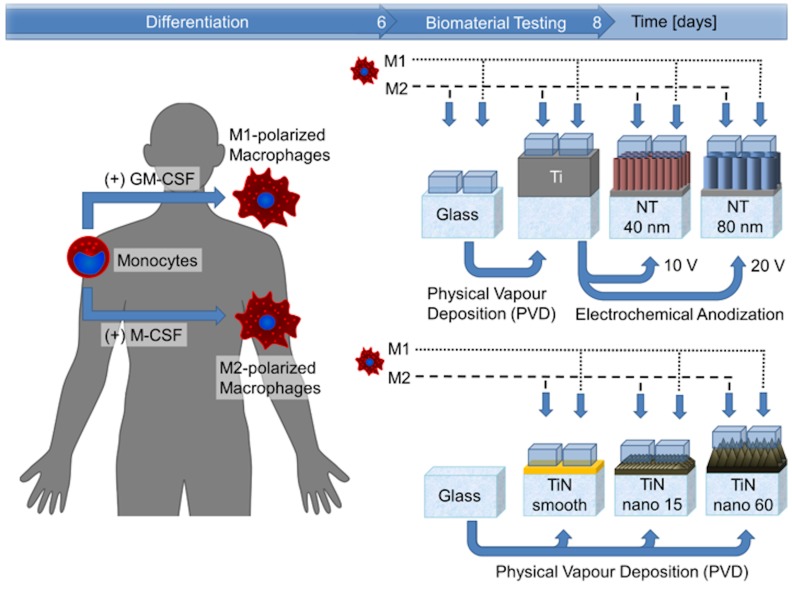
Scheme of the study design. Study design for the testing of two different types of nanostructured surfaces, with glass and suitable smooth coatings as control surfaces.

To ensure standardized test conditions, a tailored test chamber was used (see supplementary Figure S1). In this test chamber, macrophages of both subtypes were seeded on (I) glass, (II) Ti, and (III) nanotubular Ti in a cell density of 6 × 10^4^ cells per cm^2^ at a total medium volume surface ratio of 0.22 ml per cm^2^ in RPMI GlutaMax supplemented with 10% heat-inactivated fetal calf serum. All samples were incubated for 48 h at 37 °C and 5% CO_2_ conditions. The medium was harvested at 48 h and centrifuged at 10,000 × g_0_ for 5 min. The same conditions and the test chambers were also used to analyze the TiN coatings, with cells seeded on (I) glass, (II) smooth TiN, and (III) fractal TiN surfaces. The design of the complete study is presented in Figure 1.

The experiments in the first part of the study, including four different surface types (two NT surfaces, glass and Ti), were consecutively performed with cells from five human donors. Three samples were tested for each combination of macrophage polarization and surface type, leading to a total number of 24 analyzed samples per donor. 

In the second part of the study, the experiments including four different surface types (glass control and three TiN surfaces) were performed with cells from three human donors. The number of samples per donor were composed in the same way as in the first part of the study.

### 2.8. Immunohistochemical Staining

A standard protocol was performed for immunohistochemical staining on the biomaterial surfaces, as described in [3]. Briefly, cells were fixed with 4% paraformaldehyde (Carl Roth, Karlsruhe, Germany) for 10 min at room temperature. 2.5% Phalloidin Alexa Fluor 555 (A34055, Invitrogen, Carlsbad, CA, USA) in phosphate buffered saline plus 1% bovine serum albumin (Applichem, Darmstadt, Germany) was incubated for 20 min. Fluoromount-G™, with DAPI (4′,6-diamidino-2-phenylindole, eBioscience) was used for mounting. Antibody against intercellular adhesion molecule CD54 (ICAM-1, AH55411, Invitrogen) allowed visualization of cellular adherence of cells to biomaterial surfaces. The images were captured on a confocal laser scanning microscope (TCS SP8, Leica Microsysteme Vertrieb GmbH, Wetzlar, Germany). Background subtraction and contrast enhancement was equally performed on all images (ImageJ 1.49m, National Institutes of Health, Bethesda, MD, USA). Fluorescent images of macrophages on TiN surfaces were captured on a fluorescence microscope (Axio imager M1, Carl Zeiss, Göttingen, Germany) and were only stained against DAPI and Phalloidin.

### 2.9. SEM Preparation of Cell-Seeded Materials

After 48 h of culture, cells were washed with PBS- for four times, fixed in 6% glutardialdehyde in PBS- at 4 °C for 15 min and washed five times with chilled PBS for 5 min each. Samples were dehydrated in an ascending acetone series at room temperature. After critical point drying (Critical Point Dryer CPD 030, Bal-Tec, Witten, Germany) and coating with 2 nm of platinum (EM ACE600, Leica, Vienna, Austria), samples were analyzed by SEM (Zeiss CB 340).

### 2.10. Image Analysis for Morphological Studies

Images were analyzed using ImageJ (1.49m, National institutes of Health). For the assessment of the cell area and aspect ratio, at least 100 cells per cell type and surface were analyzed, therefore, data were merged from all donors. The images for this analysis were captured on a fluorescence microscope (Axio imager M1, Carl Zeiss, Göttingen, Germany). The measured cells were grouped, with a difference in area of 12 µm^2^ between one group and the following, or a difference in aspect ratio of 0.05, respectively. Subsequently, the number of cells within each single group was counted. In the next step, the percentage of the single groups to the complete distribution within a combination of cell type and surface was calculated. Afterwards, these percentages were integrated and the results for cell area were fitted using a sigmoidal fit, while the results for the aspect ratio were fitted using an asymptotic fit. The derivative of these fit functions then displays a probability density for the covered cell area, and aspect ratio, respectively. 

## 3. Results

### 3.1. Characterization of Nanotopographical Coatings 

**Figure 2:**
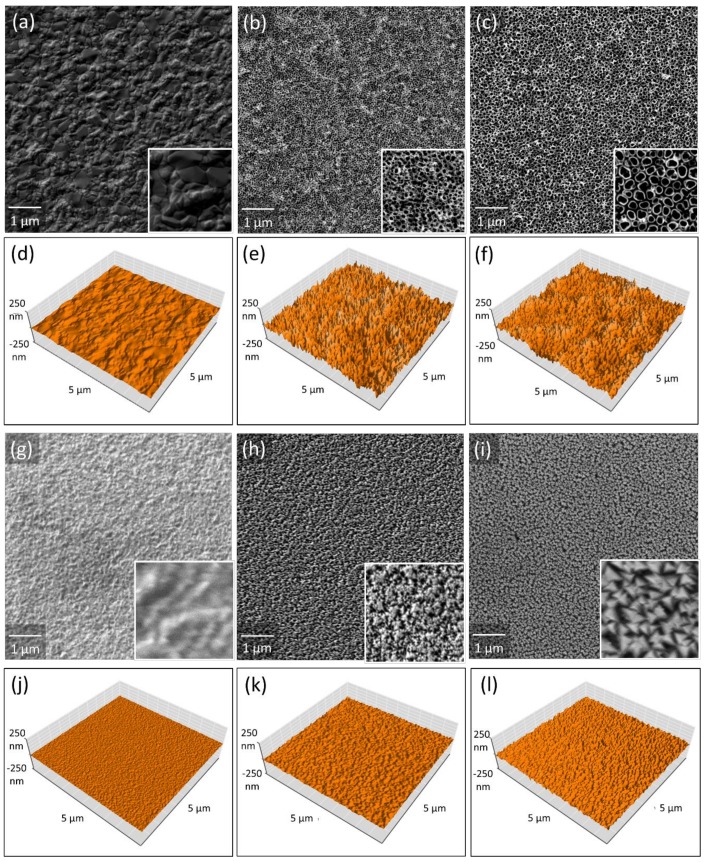
Surface characterization. (**a**) Scanning electron microscopy (SEM) picture displaying the smooth, mirror-like Ti film, deposited on a glass substrate at a substrate temperature of 200 °C; (**b**,**c**) TiO_2_ NTs generated by 2h-anodization of the Ti coatings at 10 and 20 V in an H_3_PO_4_/HF electrolyte. AFM scans of 5 µm × 5 µm areas in 3D view; (**d**) untreated Ti film deposited on a pre-heated glass substrate; (**e**,**f**) TiO_2_ NT arrays generated by anodization with voltages of 10 and 20 V in H_3_PO_4_/HF electrolyte—Figure 2 (**a**–**f**) reprinted with permission from [36]; (**g**) Smooth TiN coating produced with the substrates heated to 200 °C; (**h**,**i**) Nanorough TiN coatings deposited on unheated substrates with deposition times of 15 (TiN 15), or 60 (TiN 60) minutes, respectively. Small inserts depict higher magnification images with areas of 1 µm × 1 µm; (**j**) Atomic force microscopy scans of 5 µm × 5 µm areas in three-dimensional (3D) view of smooth TiN coating and (**k**,**l**) nanorough TiN coatings.

The nanotubular samples were initially coated with an approximately 4 µm thick Ti layer by PVD. SEM revealed that a 4 h coating of the glass substrates produced a Ti coating with a dense surface (Figure 2a). The Figure 2b,c show the Ti films after a 2 h anodization process with increasing voltages of 10 and 20 V in an electrolyte containing phosphoric and hydrofluoric acid (1M H_3_PO_4_ + 0.2 wt % HF). Increasing anodization voltage led to growing NT inner diameters from 49.5 ± 6.1, to 88.5 ± 8.5 nm and simultaneously growing NT wall thicknesses from around 9 nm to around 15 nm [36]. Alike the Ti layers, all TiN samples exhibited dense homogeneous surfaces (Figure 2g–i). While the coating produced on a heated substrate exhibited almost no topographical features (Figure 2g), coatings produced without external heating showed pyramid-like nanostructures that were growing in size with extending deposition time (Figure 2h–i). 

The nanotubular and untreated coatings were additionally investigated by AFM. Figure 2d–f displays three-dimensional (3D) views of the Ti coated glass slides and the nanotubular samples, while Figure 2j–l presents 3D views of the TiN coatings. Figure 1e shows the surface of the untreated Ti coating with a dense and smooth surface. The anodization in electrolytes containing hydrofluoric acid significantly increased the roughness of the treated surfaces (Table 2), whereby this effect was enhanced for the higher anodization voltage of 20 V (Figure 2d–f) [36]. The AFM analysis further demonstrated that the TiN control surface generated on a heated substrate had the smoothest surface of all examined samples (Figure 2j). The roughness of the TiN coatings showing pyramid-like nanostructures significantly increased, compared to the smooth control surface, and also increased with extending deposition time and size of the nanostructures. 

Contact angle measurements were performed to investigate the wettability of all sample surfaces (Table 2). A contact angle of nearly 55° revealed a modestly hydrophilic behavior of the untreated Ti coating. For the nanotubular coatings, the determination of a static contact angle was not possible, due to their superhydrophilic behavior, which led to a total spreading of the water droplet within tenths of seconds after the droplet touched the nanostructured surfaces [36]. 

The smooth TiN and TiN 15 surfaces exhibited contact angles comparably, but slightly higher than the smooth Ti coating. The TiN coating with a more pronounced nanostructure (TiN 60) showed more hydrophobic properties compared to the smooth coatings.

**Figure 3:**
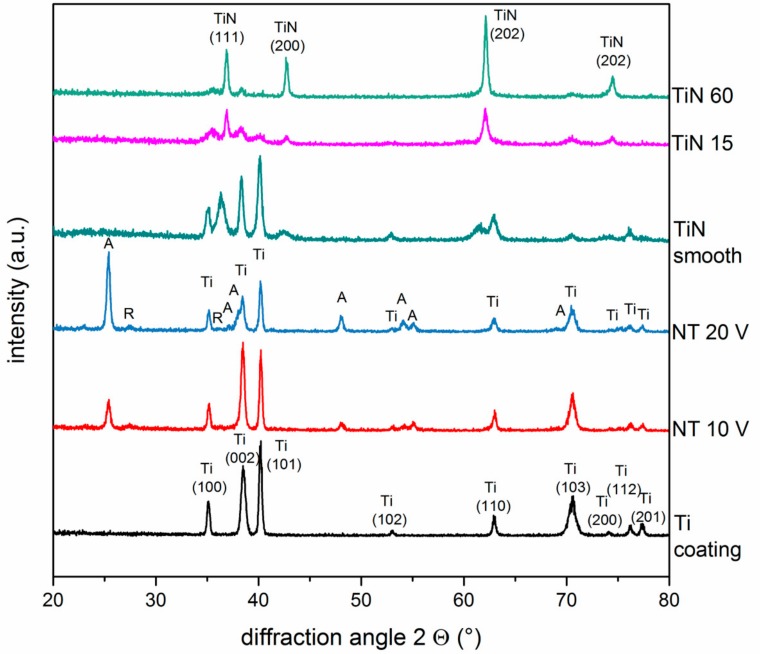
X-ray diffraction (XRD) analysis. X-ray diffraction patterns of an untreated Ti layer, smooth and nanorough TiN coatings on glass slides, as well as the TiO_2_ nanotube arrays after annealing at 450 °C for 3 h. The different phases are marked as follows: A: anatase, R: rutile, Ti: Ti coating, TiN: TiN coating. Ti and TiN lattice planes are marked with the corresponding Miller indices.

The crystallographic properties of the samples were determined by X-ray diffraction using grazing angle geometry. Figure 3 displays the diffraction patterns of the as-deposited Ti layer, as well as the pattern of the nanotubular samples after annealing. The peaks in the pattern of the as-deposited Ti layer could be attributed to the hexagonal structure of the crystal lattice of Ti and revealed a random crystal structure without a preferred orientation. The nanotubular arrays, which were amorphous in crystal structure prior to annealing, underwent transformation into anatase phase with only a minimal portion of detectable rutile after crystallization at 450 °C in a 3h-annealing process. While the increase in anodization voltage led to a significant decrease of the Ti signal, the increasing anodization voltage simultaneously caused a significantly increased signal intensity of the anatase peak [36].

The diffraction patterns of the TiN coatings showed an overlay of the Ti base layer and the TiN top layer. Diffraction peaks, attributed to the Ti base coating, were assigned to a Ti hexagonal crystal lattice, whereas the TiN peaks could be attributed to a δ-TiN lattice with fcc crystal structure. The diffraction pattern of the smooth TiN coating exhibited broader peaks compared to the surfaces showing pyramid-like nanostructures. In the case of the TiN 15 and TiN 60 coatings, the intensity of the TiN peaks increased with extending deposition time, whereas at the same time the signal intensity attributed to the Ti base layer decreased.

### 3.2. Flow Cytometry Confirms Robust Polarization of Macrophages

Monocytes were isolated from peripheral blood, and differentiated into macrophages. Flow cytometry measurements ensured the required cell and phenotype of obtained cell populations. Following in vitro differentiation, both M1 and M2 subtype macrophages were positive for CD14, CD68, and CD206. For further experiments, only cells from donors with purity of more than 90% were used for further experiments. The presence of CD80 and CD163 was analyzed to discriminate between the two subtypes (Figure S2). As expected, macrophages polarized to M1 phenotype exhibited a high percentage of CD80-positive cells, whereas CD163-positive cells were absent. In contrast, M2-polarized macrophages were positive for CD163 and negative for CD80.

### 3.3. Cell Morphology of M1 and M2 Phenotype Macrophages Following Cell-Surface Interaction with Tubular Nanostructures

**Figure 4:**
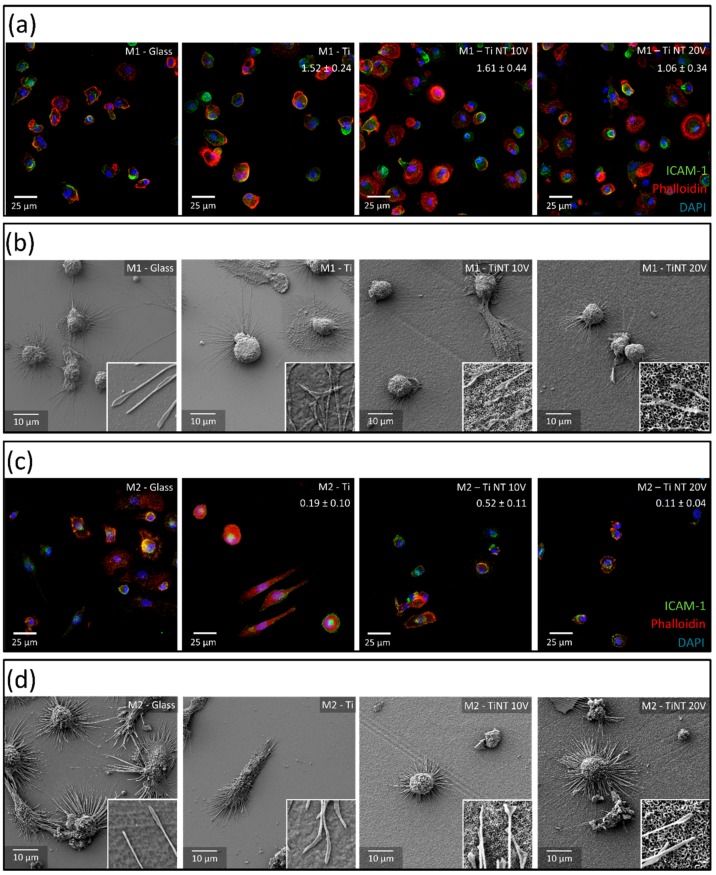
Morphological analysis of biomaterial-resident macrophages after 48 h of culture. Following 48 h of culture, cell morphology of M1 macrophages was visualized by (**a**) confocal microscopy and (**b**) SEM imaging. Images are exemplarily shown for one of five donors. The small inserts in the SEM-images show a higher magnification image with an area of 2 µm × 2 µm. (**c**) Confocal images of M2 macrophages and the (**d**) respective SEM analysis facilitate the comparison of both subtypes. Glass, Ti coating, and Ti nanotubes (NT) produced by anodization of the physical vapor deposition (PVD) coatings at 10 and 20 V in an H_3_PO_4_/HF electrolyte for 2 h. The cell numbers counted for each material and phenotype in relation to the glass control samples are inserted in the respective images.

After 48 h of culture on the generated Ti and nanotube surfaces and glass as a control, material-resident cells were stained with DAPI, Phalloidin and ICAM-1 (Figure 4a,c) demonstrating a material-specific cell pattern. On glass surfaces, both phenotypes exhibited a rather round cell morphology, whereas on Ti surfaces most M2 macrophages took an elongated shape. The shape of M1 macrophages on Ti was comparable to their shape on glass. On the nanotubes, the M1 macrophages’ morphology was similar to that on glass and on unstructured Ti. In contrast to M1 macrophages, both nanotubular topographies had a strong impact on M2 phenotype macrophages. The elongated cell morphology found on flat Ti turned to a round cell shape with a cytoplasm closely organized around the nucleus. 

For each material surface, 12 images of DAPI and Phalloidin stained cells were randomly selected for image automated analyses. The cell numbers counted for each material and phenotype normalized to glass are inserted in the respective images in Figure 4. Cell counting revealed that in comparison to glass, higher cell numbers of M1 phenotype macrophages were detected for the generated non- and nano-structured coatings (Ti: 1.52 ± 0.24; Ti-NT 10V: 1.61 ± 0.44; Ti-NT 20V: 1.06 ± 0.34), whereby, this effect was reduced with increasing nanotube diameter (Figure 4). In contrast, significantly less M2 phenotype macrophages were found on Ti and the Ti nanotube surfaces, compared to the control experiment (Ti: 0.19 ± 0.10; Ti-NT 10V: 0.52 ± 0.11; Ti-NT 20V: 0.11 ± 0.04). Thereby, lowest cell density was found for nanotubes exhibiting the highest diameter.

Since information obtained by confocal microscopy is limited to cell morphology, the cell-material interaction was assessed by SEM (Figure 4b,d). In the depicted SEM images, typical cell morphologies found in fluorescent images for M1- (Figure 4a) and M2-macrophages (Figure 4c) on the different material surfaces were confirmed. In the low magnification SEM images, cells were attached to apparently flat surfaces. To better compare between the scale of the topography and the size of the cells, higher magnification of the macrophage filopodia are included as small insets. Hereby, a close interaction between the cytoplasmic sprouts and the surface structure was detected for all surfaces.

### 3.4. Statistical Analysis of Cell Morphology of M1 and M2 Phenotype Macrophages Reveals a Phenotype-Specific Cell-Material Interaction with Nanotubular Surfaces

**Figure 5:**
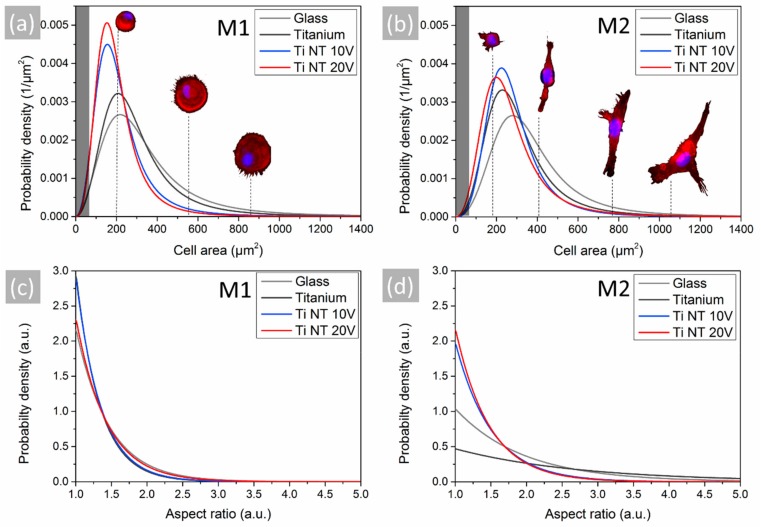
Statistical analysis of cell morphology. Probability density of (**a**,**b**) cell area and (**c**,**d**) aspect ratio for the M1- and M2-polarized macrophages on glass, Ti and the Ti nanotubes (NT) surfaces obtained from fluorescence microscopy images. Data were merged for all five donors. Images of cells typically found at a size indicated by the dashed lines are inserted into the graphs to facilitate conceivability. The darkened parts in graph (**a**,**b**) indicate the range of cell size where no cells could be detected.

For the statistical analysis of the cell morphology on the different surfaces, fluorescence microscopy images were analyzed regarding the area covered by single cells, as well as the respective aspect ratio. After evaluating more than 100 cells per surface for each cell phenotype, the results of the obtained cell surface area distribution were fitted using a sigmoidal function (R2 > 0.99), thereby generating a probability density for the covered cell area (Figure 5a,c). In addition, a probability density for the respective aspect ratio of these cells was generated by using an asymptotic function (R2 > 0.94; Figure 5b,d). Interestingly, we could find cell-specific patterns for the cell area and aspect ratio on different materials. In particular, the M1 subtype typically exhibited a round shape on all tested surfaces. While, cell size decreased on nanostructured surfaces compared to the flat surfaces, M1 macrophages generally covered a larger area than M2. In contrast to M1 macrophages, a material-specific difference in morphology was obvious for the M2 phenotype. The probability for smaller and rounder cells was highly increased on the nanostructured surfaces compared to the flat surfaces made of Ti or glass; whereby this impact was slightly stronger with increasing nanotube diameter. The highest probability density for elongated cells could be found on the sputtered Ti surfaces. 

### 3.5. Cell Morphology of M1 and M2 Phenotype Macrophages on TiN Surfaces Following Cell-Surface Interaction

**Figure 6:**
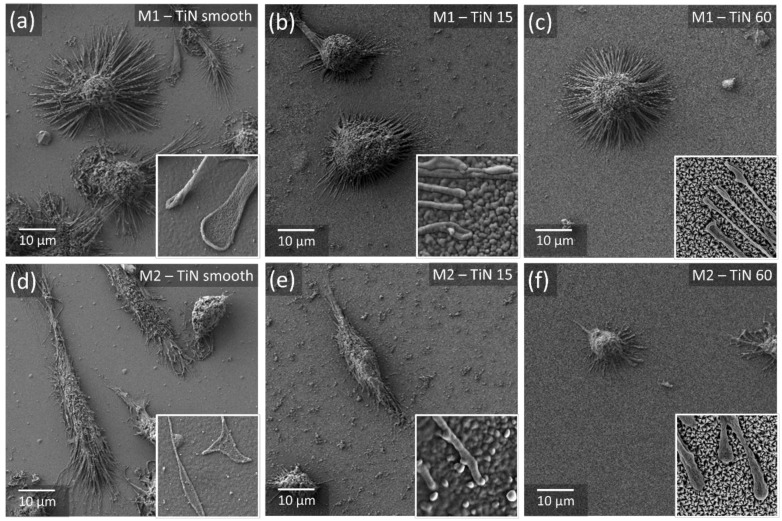
Morphological analysis of biomaterial-resident macrophages. Following 48 h of culture, cell morphology of the two phenotypes (**a**–**c**) M1 and (**d**–**f**) M2 was visualized by SEM imaging. Images are exemplarily shown for one of three donors. The small inserts in the SEM-images show a higher magnification image with an area of 2 µm × 2 µm with focus on the filopodia of the cells.

In order to investigate whether the two phenotypes of macrophages react in a similar way to a different style of size-adjustable nanostructures, the same experimental procedure, as described for the nanotubes, was extended to a set of TiN samples that were generated as a smooth surface, but also equipped with two grades of pyramid-like nanostructures. Therefore, the cells from three new donors were used. SEM images of macrophages on the TiN surfaces are presented in Figure 6, with small inserts in higher magnification focusing on the filopodia of the cells to provide a better impression of the size ratio between nanofeatures and cell size.

The material-resident cells on the three different TiN surfaces and the glass control samples were stained with DAPI and Phalloidin (Figure S3), thereby exhibiting again a material-specific cell pattern. 

The M1 macrophages showed a round cell morphology on all the tested surfaces, with only their size obviously shrinking when the underlying surface had highly pronounced nanostructures as the TiN 60 surface. More distinct contrasts in cell morphology could be found for the M2 macrophages. While cells of M2 phenotype on the smooth TiN coating were elongated to a great extent, only small cells of round shape could be found on the highly nanostructured TiN 60 surfaces. In contrast to the TiN 60 coatings, a significant amount of M2 macrophages with elongated shape could be found on the TiN 15 surfaces. 

As described in the section above, cell numbers per image were counted and normalized to the respective number of the phenotypes on the glass control samples. These normalized cell numbers are inserted in the respective images in Figure S3. The number of cells of M1 phenotype did not significantly differ from the number of cells counted on the glass control samples, which is indicated by a normalized cell number value close to 1 (TiN smooth: 1.24 ± 0.61; TiN 15: 1.08 ± 0.45; TiN 60: 0.83 ± 0.16). However, the number of cells of M2 phenotype decreased significantly with an increasing degree of nanostructure. Where the normalized cell number was again close to 1 for the smooth TiN surface (0.83 ± 0.48), it was more than halved for the TiN-15 coatings (0.43 ± 0.06). Compared to the glass control surfaces, only around 20% of the number of cells could be detected on the TiN 60 surfaces (0.23 ± 0.10).

### 3.6. Statistical Analysis of Cell Morphology of M1 and M2 Phenotype Macrophages on TiN Surfaces Reveals a Phenotype-Specific Cell-Material Interaction

**Figure 7:**
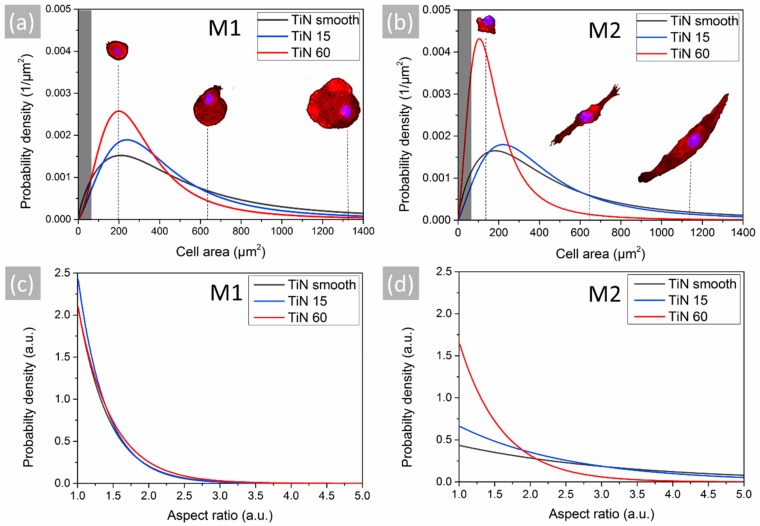
Statistical analysis of cell morphology. Probability density of (**a**,**b**) cell area and (**c**,**d**) aspect ratio for the M1- and M2-polarized macrophages on smooth and nanorough TiN surfaces obtained from fluorescence microscopy images. Data were merged for all three donors. Images of cells typically found at a size indicated by the dashed lines are inserted into the graphs to facilitate conceivability. The darkened parts in graph A and B indicate the range of cell size where no cells could be detected.

The fluorescence microscopy images were in addition used to perform statistical analyses of the area covered by single cells and their respective aspect ratio on the smooth and nanostructured TiN coatings (Figure 7). After evaluating the results of the three different coatings we could find again cell-specific patterns for the cell area and aspect ratio of the macrophages.

The statistical analyses of the M1 phenotype revealed that the cell size distribution on the smooth TiN coating was the most homogenous of all tested samples (Figure 7a), with a significant number of wide-spread cells. Compared to the flat TiN surface, the size of the cells was decreasing on the TiN 60 coating. In contrast, the size distribution of M1 cells on the TiN 15 coatings was comparable as homogenous to the smooth surface (Figure 7a). Similar to the results of the experiment with nanotubular samples, the cells of M1 phenotype particularly exhibited a round shape (Figure 7c). Another similarity to the previous experiment could be found for the statistical analysis of the M2 phenotype (Figure 7b,d). In particular, in the samples showing a higher degree of nanotopography, the TiN 60 coatings, the probability density of small and round cells was the highest of all tested samples in both experiments. In contrast to the small and round cells found on the TiN 60 layers, both the probability density for larger size and especially an elongated shape was highly increased on the smooth and moderately structured TiN surfaces. Thus, in this experiment only the sample with a higher degree of nanotopography had a strong impact on the M2 phenotype and was even stronger than the effect of the nanotubular surfaces.

## 4. Discussion

All implanted biomaterials induce cellular and tissue responses that finally result in the encapsulation of the foreign body [1]. In this wound healing process, macrophages play a pivotal role. Macrophages originate from monocytes that rapidly differentiate when recruited in the context of inflammation [39,40]. At the wound site, macrophages exhibit a spectrum of polarization ranging from pro-inflammatory to anti-inflammatory and pro-healing character, depending on their specific phenotype. Hereby, M1-polarized macrophages are associated with pro-inflammation, whereas M2 macrophages are the dominant phenotype in the remodeling phase of wound healing [41]. Thus, the impact of a biomaterial on a specific macrophage phenotype is of interest for the improvement of implant materials. A possibility to control the macrophage polarization is to equip an implant with a nanotopography, which can be generated in many different styles, such as nanotubes or pyramid-like fractal nanostructures. The first can be generated on a variety of different implant materials by coating the material with Ti, and subsequently introduce the nanostructure by electrochemical anodization, the latter can be directly produced by a PVD process. Due to the opportunity to fabricate tubular and fractal surface structures, with tunable dimensions in the nanoregime, by simply varying easy-accessible process parameters, such as the anodization voltage or PVD process time. Both PVD and electrochemical anodization achieve a high effectiveness and reproducibility. The controlled fabrication and reproducibility is a prerequisite to studying the impact of different nanostructure dimensions on material-resident cells [42].

The parameter setting on heated glass substrates, used for the PVD coating process, facilitated fine-grained and smooth coatings suitable for the fabrication of ordered nanotube arrays. Furthermore, higher substrate temperatures increased the temperature stability and adhesion between the deposited film and the substrate. This prevented the nanotubular structures from delamination or damaging by higher temperatures used during the annealing process. A combination of diluted H_3_PO_4_ and HF and was used for the anodization process. This sort of electrolyte provides a high degree of control regarding the geometry of the NT arrays, especially the diameter of the nanotubes, an effect attributed to the buffering potential of H_3_PO_4_ [37,43]. The produced NT arrays exhibited well-defined tubes, whereby, the average nanotube diameters increased with rising anodization voltage and seemed to be in good accordance with the tube diameters, reported by other groups who anodized bulk titanium at the same range of voltages in electrolyte mixtures of H_3_PO_4_ and HF [37]. As expected, the AFM analysis of the nanotubular surfaces exhibited much rougher surfaces compared to the mirror-like Ti coating, whereby larger tube diameters also led to increased surface roughness. The detected increase in the roughness values could be clearly attributed to the tubes’ nanoscale roughness, since the generation of highly reproducible coatings, together with a subsequent defined pre-etching step, minimized the variations of the surface roughness of the underlying PVD coatings. 

The nanotube arrays, generated by anodization in H_3_PO_4_/HF electrolyte, exhibited superhydrophilicity. However, this beneficial property regarding cell adhesion and protein adsorption appeared to be independent of the diameter, and length of the nanotubes, respectively. The increased intensity of the anatase peak in the X-ray diffraction pattern recorded for rising anodization voltages, as well as the simultaneously observable decline of the Ti signal, that was associated with the remaining underlying coating, can be attributed to an increasing thickness of the NT array with rising voltage, an effect that could also be observed at the anodization of bulk titanium [35]. A typical finding in the diffraction patterns of NT arrays annealed at 450 °C is also the observed phase mixture with a high ratio of anatase to rutile [42]. 

In contrast to the nanotube arrays, the TiN coatings could be directly produced via PVD process [30]. Two sets of coating parameters were used for the fabrication of TiN surfaces. First, using heated substrates, a very smooth coating with roughness values even lower than the pure Ti coating was generated. The second set of parameters without the application of the substrate heating was used to generate surfaces with pyramid-like fractal nanopatterns. The extension of the deposition time led to the growth of the pyramid-like structures, and thus, resulted in larger and well-defined sharp nanostructures. The formation of the self-assembled nanostructures also caused a significant increase of the coatings roughness compared to the as-deposited smooth TiN, whereby the growth of the fractals’ size was also accompanied by a further roughening of the surface. Regarding the wettability of the TiN samples, the contact angle measurement revealed that both the smooth TiN coating and the coating with smaller fractals exhibited contact angles close to the pure Ti coating. Only the larger fractals caused a moderately shift to a more hydrophobic behavior, however the measured angles still were in the hydrophilic regime. 

The XRD analysis detected for all samples both signals from the underlying Ti coating and the top layer of TiN. Due to the thickening of the TiN top layer, the signal intensity attributed to the underlying Ti coating decreased, while the intensity of the TiN signals increased with the extension of the deposition time. The signals detected in the XRD analysis, which could be attributed to the different TiN layers could be assigned to a δ-TiN lattice with fcc crystal structure [29]. 

Nanotubular topographies have a strong impact on monocyte differentiation into macrophages. In vitro, it was observed that monocytes spontaneously differentiate into macrophages on nanotubular Ti surfaces, and that small nanotube diameters promote polarization towards M2 phenotype, whereas large nanotube diameters result in polarization towards M1 phenotype [25]. However, in addition to surface properties, also biochemical conditions are known to instruct macrophage polarization [44,45]. For example, key mediators of first line humoral surface interaction, e.g., blood plasma proteins modulate cell adhesion, blood coagulation, and complement activation. Moreover, the release of alarm signals to the extracellular space, induced by tissue injury, stimulates the migration of immune cells from the vasculature to local implant region [38], and thus, it has not been clarified yet whether the polarization effect of nanotubes on differentiating monocytes found in vitro can still be detected in a physiological wound scenario. Additionally, at a wound site without a biomaterial, monocytes rapidly differentiate into macrophages [40], and it is unclear how differentiated human macrophages are affected when arriving at a nanotubular topography. 

A transition between both phenotypes cannot be excluded since the identity of macrophages has been described as rather a continuum than a distinct M1 or M2 phenotype [46]. Nevertheless, even if a transition occurs, for example from M2 to M1, the cell characteristics of putatively switched M2 macrophages strongly differ from initially M1-polarized cultures. Although, a switch between macrophage phenotypes is theoretically possible, in our previous studies we could not detect a switch after only 48 h of culture, especially without external stimulation of the macrophages [3,4].

Following verification of phenotype, M1- and M2-polarized macrophages were cultured on the nanotubular surfaces and respective controls; glass and a mirror-like Ti coating. SEM revealed that, on all surfaces, both phenotype types attached and established an intense interaction with the surface by the formation of cytoplasmic sprouts. Immunohistochemical imaging of cell morphology indicated a phenotype-specific impact of the nanotubes, which was confirmed by statistical analysis of cell surface areas and aspect ratio. While on Ti and nanostructured Ti M1 macrophages exhibited comparable morphological parameters, nanostructured Ti-impaired cell spreading of M2 macrophages. Interestingly, unstructured Ti surfaces promoted elongation of M2 macrophages, which is associated with migration and an increased cell activity [8]. Reduced cell numbers and cell spreading of human-derived macrophages on nanotube surfaces could also be demonstrated by Smith et al. [47]. However, it must be noted, that there are two major differences between the studies: (I) Human monocytes there were directly cultured on the materials; and (II) the nanotubes were prepared in a diethylene glycol (DEG) electrolyte, that significantly increased the space between single nanotubes, which resulted in a quite different surface structure.

In an attempt to find out whether the two macrophage phenotypes express a similar morphological reaction when exposed to another type of scalable nanostructure, the M1- and M2-polarized cells were cultured for 48 h on TiN layers, equipped with two different sizes of pyramid-like patterns, with glass and a smooth TiN coating as controls. The SEM analysis of the samples could demonstrate the attachment of the cells to the surface and the establishment of material interaction by cytoplasmic sprout formation. According to the protocols established for the investigation of Ti-NT surfaces, images were taken by fluorescence microscopy and then statistically analyzed. The analysis of these images revealed remarkable similarities between the two series of experiments using different sets of surfaces. 

Similar to the previous experiments, the M1 macrophages exhibited a rather round shape on all surfaces. However, the general impact of the TiN nanostructure on the M1 phenotype was lower compared to the Ti-NT surfaces. The cell areas covered by the single cells, were comparably larger and more evenly distributed on all tested TiN surfaces than in the NT experiments. On the smooth TiN surface, which exhibited roughness values close to glass, and therefore, the surface with the lowest roughness value of all generated coatings, the M1 macrophages were wide-spread and had the most homogeneous distribution regarding the area covered by single cells. Remarkably, M2 macrophages, cultured on smooth TiN, had an almost identical probability density regarding the distribution of the aspect ratio compared to unstructured Ti coatings, which means that on both coatings with a different surface chemistry, similarly high ratio of elongated cells could be found. However, the size of the M2 cells was significantly larger than on the smooth Ti coating, which renders this surface, together with the fact that the measured cell numbers were close to the glass control samples, possibly the best accepted coating for the M2 phenotype. 

Although, TiN-60 coatings only seem to have limited influence on the M1 phenotype, these sharp pyramid-like nanopatterns had a significant impact on the M2 phenotype, indicated by drastically reduced cell numbers and cell sizes. This finding is in very good agreement with the results for the NT surfaces, whereby the impact of the TiN 60 coating on the M2 cell size seem even stronger than the impact of the large diameter nanotubes. 

The most obvious difference between both experiments can be found when comparing the two samples that were equipped with small diameter nanotubes (NT 10V) and smaller fractal patterns (TiN 15), respectively. Where the cells of both M1- and M2-phenotype had more or less similar morphologies on NT 10V and NT 20V surfaces, the macrophages’ morphologies of both phenotypes on the TiN 15 coatings closely resembled the morphologies on the smooth TiN coatings. Thus, even for the M2 macrophages that were quite sensitive to both tube sizes and the larger fractals, there seems to be a threshold regarding the size of the nanostructures that could actually limit their spreading and cell activity. 

Low cell numbers of M2 macrophages on nanotube and fractal surfaces, compared to M1 macrophages and impaired cellular activity led to the assumption that the nanostructured coatings are less tolerated by M2-polarized macrophages than smooth Ti or TiN coatings. Whereas, the impact of the investigated nanostructures on M1-polarized macrophages was limited. An exception to this were the TiN-15 surfaces, which probably exhibited nanostructures small enough to be more tolerable for the M2-phenotype.

An explanation for the phenotype-specific impact of the nanotubular structures could be that M1 and M2 macrophages differ regarding their complexity and function. While, the synthesis of pro-inflammatory cytokines is an attribute for M1 macrophages, M2 macrophages are equipped with several receptors for endogenous and foreign body molecules [48], which indicates a more sensoric function. Moreover, in contrast to M1 macrophages, the secretome of M2 macrophages comprises more proteins, M2 macrophages differentiate in different M2 subtypes [39], the M2 phenotype modulates more processes in the FBR [41], and M2 polarization is based on a stronger interaction with various immune cells [41,49,50]. Thus, the complex range of function could be an explanation why M2 macrophages respond more sensitively to their nanoenvironment. 

## 5. Conclusions

The results of our study, revealed remarkable differences regarding the impact of the nanotopography on the different phenotypes. M1-polarized macrophages were to a great extent unaffected regarding adherent cell numbers and morphology, while major differences could be found for the M2 phenotype. The identified phenotypic response of macrophages bears potential to modulate the early immunological response, e.g., by leaving the M1 phenotype largely unaffected, while restricting the adherence and activity of M2 macrophages.

## Figures and Tables

**Table 1:** Parameters of the PVD processes for the deposition of Ti, as well as smooth and nanorough TiN.

Sample	Substrate	Base Coating				Top Coating			
	Temperature	Process Gas		Power	Time	Process Gas		Power	Time
-	-	Ar	N_2_	-	-	Ar	N_2_	-	-
-	(°C)	(sccm)	(sccm)	(W)	(min)	(sccm)	(sccm)	(W)	(min)
Ti smooth	200					137	0	400	240
TiN smooth	200	137	0	500	30	137	2	800/600	2/18
TiN 15	n.a.	137	0	500	30	137	2	800/500	2/60
TiN 60	n.a.	137	0	500	30	137	2	800/500	2/60

**Table 2:** Physical properties of the glass substrate, untreated Ti layer and the nanotubular surfaces, as well as the smooth and nanorough TiN coatings.

Sample	Nanotube	Roughness			Contact
	Diameter	Average	rms	-	Angle
	(nm)	Ra (nm)	Rq (nm)	Rz (nm)	(°)
Glass	-	1.21 ± 0.13	1.52 ± 0.15	7.57 ± 0.81	98.1 ± 1.8
Ti coating on glass	-	6.13 ± 0.67	7.61 ± 0.58	35.89 ± 5.72	54.8 ± 3.0
NT 10 V	49.5 ± 6.1	19.45 ± 1.86	24.17 ± 2.81	122.47 ± 10.76	< 5
NT 20 V	88.5 ± 8.5	33.49 ± 3.89	40.81 ± 3.51	209.01 ± 19.79	< 5
TiN smooth	-	1.51 ± 0.08	1.86 ± 0.11	9.19 ± 1.27	58.8 ± 1.3
TiN-15	-	6.33 ± 0.22	7.60 ± 0.06	38.01 ± 3.59	58.0 ± 0.4
TiN-60	-	9.07 ± 1.51	11.56 ± 1.90	63.51 ± 9.29	69.9 ± 5.3

## Supplementary Materials

The following are available online at https://www.mdpi.com/1996-1944/13/5/1142/s1, Figure S1: Experimental setup for in vitro biomaterial test; Figure S2: Confirmation of macrophage phenotype prior to material testing; Figure S3: Morphological analysis of biomaterial-resident macrophages.
