# Supplementary material for: Nanotopographical Coatings Induce an Early Phenotype-Specific Response of Primary Material-Resident M1 and M2 Macrophages
Source: Materials (Basel). 2020 Mar 4;13(5):1142. doi: 10.3390/ma13051142 (PMC7084960; doi:10.3390/ma13051142)
Supplement: Supplementary file 1 [file materials-13-01142-s001.pdf]

materials

Supplementary Materials

Nanotopographical Coatings Induce an Early Phenotype-Specific Response of Primary Material-Resident M1 and M2 Macrophages

Tobias Schmitz [1,*], Maren Jannasch [1], Tobias Weigel [1,2], Claus Moseke [3], Uwe Gbureck [4], Jürgen Groll [4], Heike Walles [5] and Jan Hansmann [1,2]

[1] Department Tissue Engineering and Regenerative Medicine (TERM), University Hospital, Würzburg 97070, Germany
[2] Translational Center Regenerative Therapies (TLC-RT), Fraunhofer Institute for Silicate Research ISC, Würzburg 97070, Germany
[3] Institute for Biomedical Engineering (IBMT), University of Applied Sciences Mittelhessen (THM), Gießen 35390, Germany
[4] Department of Functional Materials in Medicine and Dentistry (FMZ), University Hospital, Würzburg 97070, Germany
[5] Core Facility Tissue Engineering, Otto von Guericke University, Magdeburg 39106, Germany
* Correspondence: tobias.schmitz@uni-wuerzburg.de

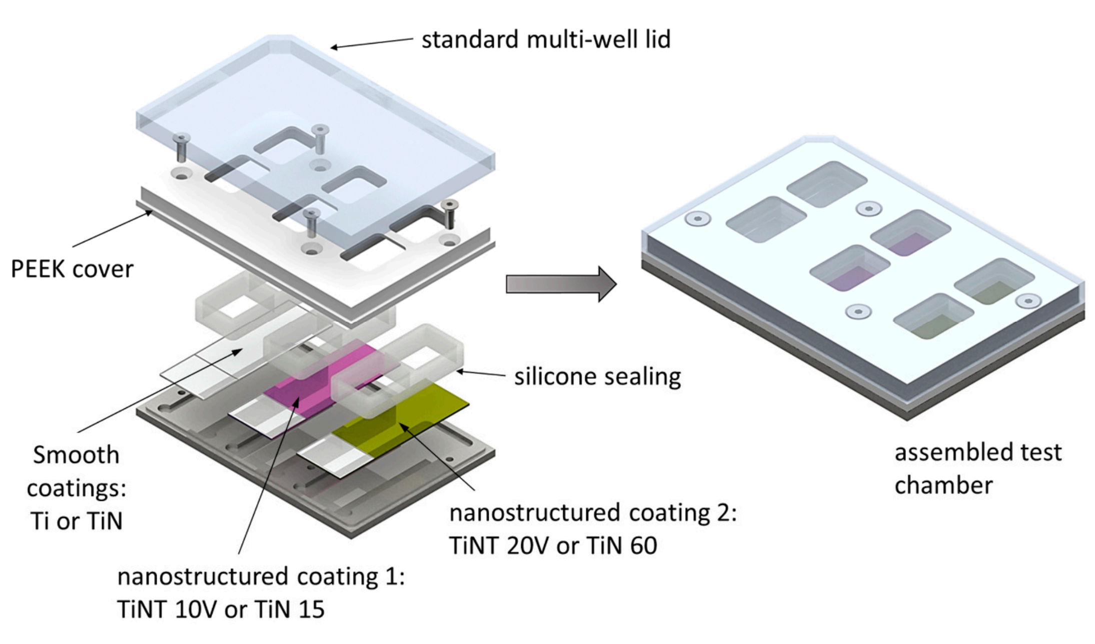

Figure S1. Experimental setup for in vitro biomaterial test. During testing, samples were transferred in a tailored test chamber. The test chamber ensured standardized testing and facilitated culture under sterile conditions in an incubator.

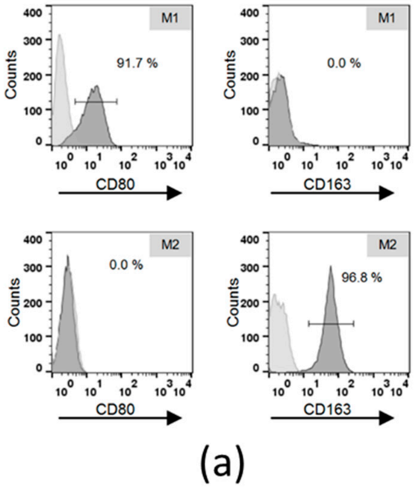

Donor	M1 CD80	M1 CD163	M2 CD80	M2 CD163
1	61.2	0	0	68.8
2	91.7	0	0	96.8
3	66.9	0	0	90.6
4	81.2	0	0	96.4
5	86.6	0	0	90
Mean	77.5±11.7	0	0	88.5±10.2

(b)

Figure S2. Confirmation of macrophage phenotype prior to material testing. (**a**) Histograms for CD80 and CD163 represent the distribution of phenotypic differentiation clusters on primary blood-derived macrophages, here exemplarily shown for one cell donor. (**b**) The percentage of positively stained cells on differentiation clusters is shown for all five donors. A high compliance between the donors was found.

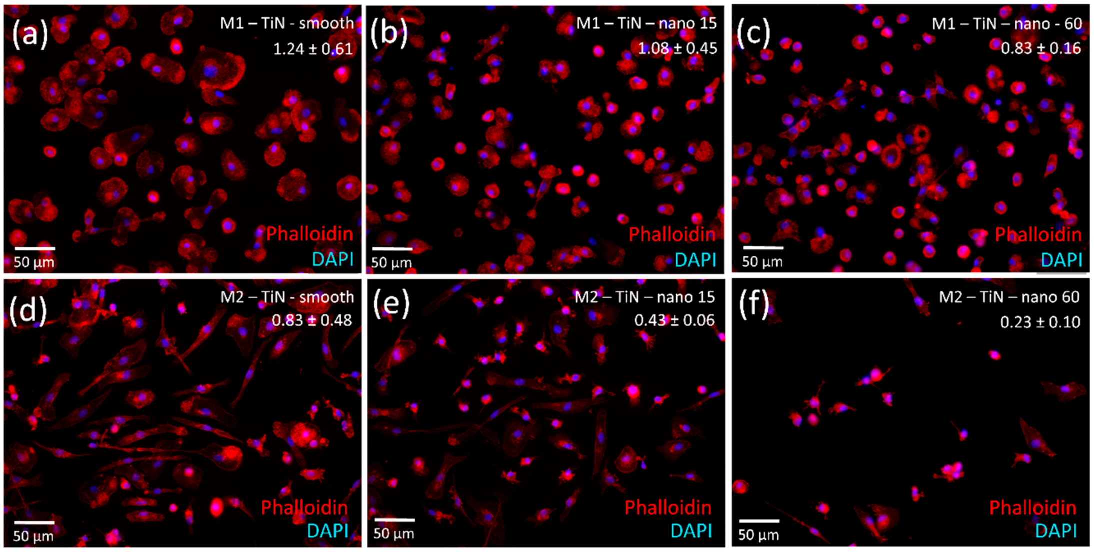

Figure S3. Morphological analysis of biomaterial-resident macrophages. Following 48 h of culture, cell morphology of the two phenotypes (**a**–**c**) M1 and (**d**–**f**) M2 was visualized by fluorescence microscopy. Images are exemplarily shown for one of three donors. The cell numbers counted for each material and phenotype in relation to the glass control samples are inserted in the respective images.

© 2020 by the authors. Submitted for possible open access publication under the terms and conditions of the Creative Commons Attribution (CC BY) license (http://creativecommons.org/licenses/by/4.0/).
